# Research and Development Strategies for Hybrid *japonica* Rice

**DOI:** 10.1186/s12284-020-00398-0

**Published:** 2020-06-08

**Authors:** Wenjing Zheng, Zuobin Ma, Mingzhu Zhao, Minggang Xiao, Jiaming Zhao, Changhua Wang, Hong Gao, Yuanjun Bai, Hui Wang, Guomin Sui

**Affiliations:** 1 Institute of Rice Research, Liaoning Academy of Agricultural Sciences, Shenyang, 110000 China; 2 Heilongjiang Academy of Agricultural Sciences, Haerbin, 1550086 China; 3 Liaoning Academy of Agricultural Sciences, Shenyang, 110161 China

**Keywords:** Hybrid *Japonica* Rice, Three-line, Two-line, Heterosis

## Abstract

The utilization of heterosis has resulted in significant breakthroughs in rice breeding. However, the development of hybrid *japonica* has been slow in comparison with that of hybrid *indica*. The present review explores the history and current status of hybrid *japonica* breeding. With the creation of *japonica* cytoplasmic male sterility and photo-thermo-sensitive genic male sterile lines, both three-line and two-line systems of hybrid rice have been created, and a series of hybrid *japonica* rice varieties have been developed and cultivated widely. At the same time, some progress has been made in genetic research of molecular mechanism for heterosis and QTL mapping for traits such as fertility, stigma exposure and flower time. In addition, genomics and transcriptome have been widely used in the research of hybrid rice, which provides a strong support for its development. Although the research on hybrid *japonica* has made many advances, there are still some restrictive problems. Based on the research and production of hybrid *japonica* rice, the prospect and development strategies of hybrid *japonica* rice are analyzed.

## Introduction

**Fig. 1:**
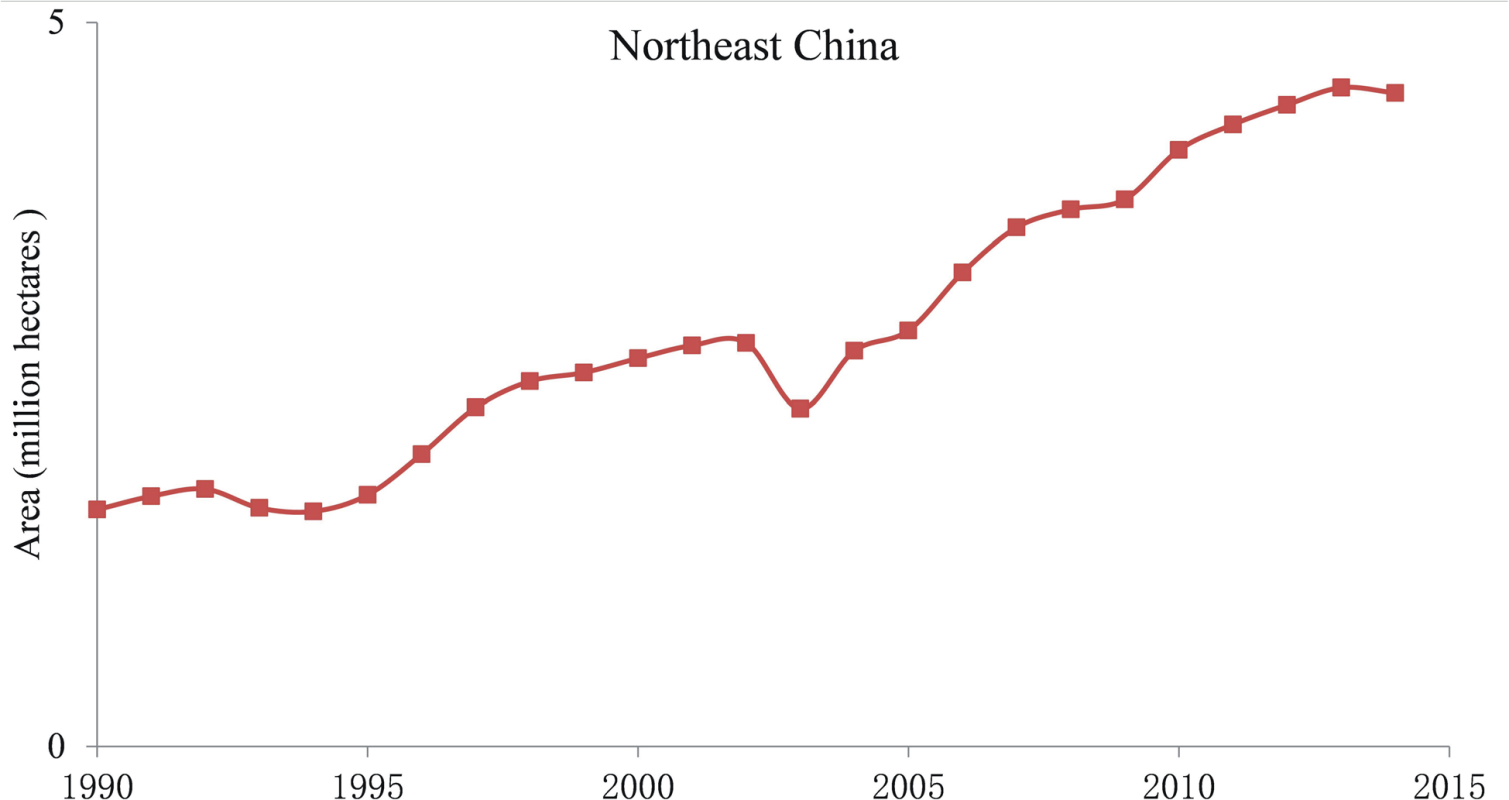
The annual planting area of *japonica* rice in northeast China from 1990 to 2015

**Fig. 2:**
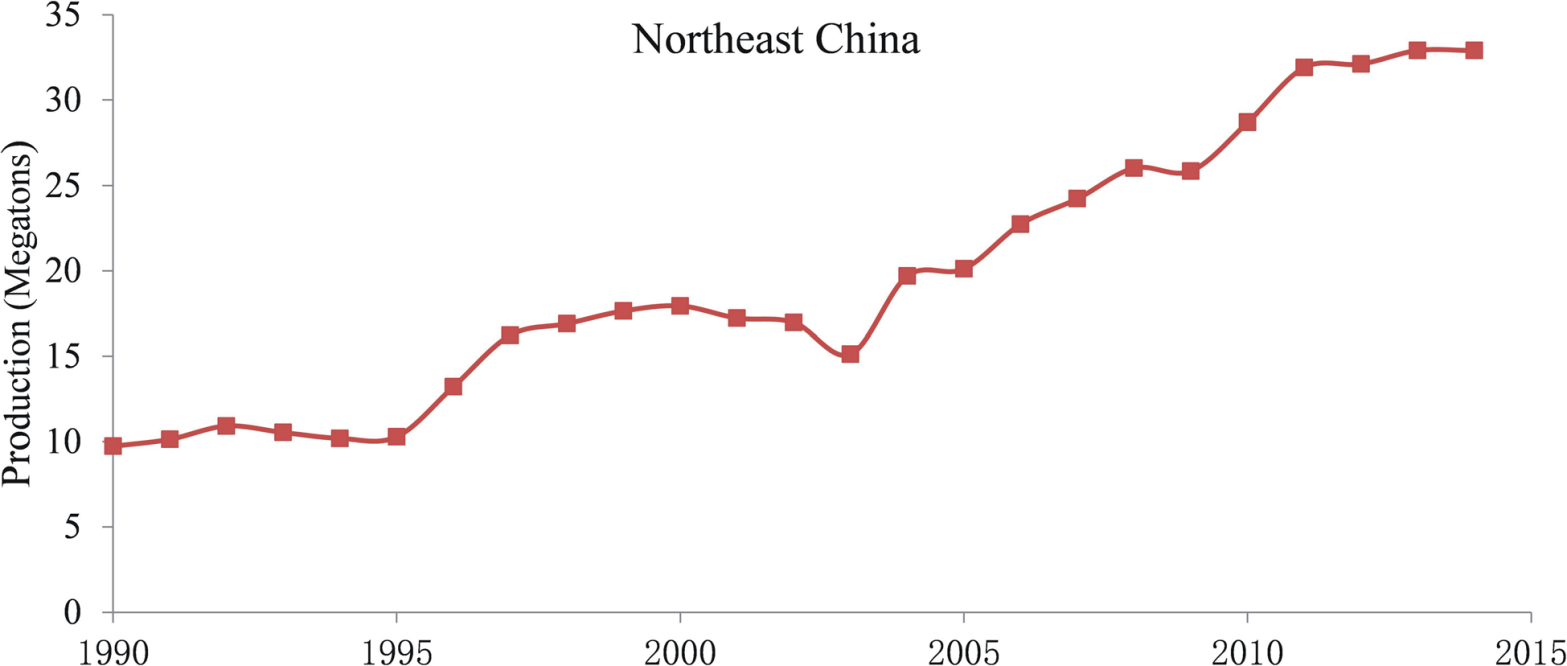
The annual production of *japonica* rice in northeast China from 1990 to 2015

Among more than 120 rice-producing countries, over 95% of them cultivate primarily *indica* varieties (Deng 2008; Fang 2005). Globally, only a few countries, such as China, Japan, South Korea, United States, Australia, and Egypt, produce and export *japonica* rice (Deng 2008). The *japonica* varieties occupy 8.8% of the rice farming area and are responsible for 14.2% of total rice production worldwide (Fang 2005). From 2007 to 2015, the total amount of rice traded on international markets increased from 32 million tons to 41.67 million tons, while the proportion of *japonica* rice trade decreased during the same time from 10.9% to 8.1%. This decrease corresponds to approximately 3.5 million tons, which is less than 5% of the amount of *japonica* rice consumed in China (Cao et al. 2018). China has the largest planting area and the highest total production of *japonica* rice in the world. Because the culinary quality of *japonica* is superior to that of the *indica* rice, the total production and demand for *japonica* rice has been increasing continuously in China, especial in Northeast China from 1990 to 2015 (Fig. 1, Fig. 2) (Tang et al. 2017). To meet the growing need, the share of the *japonica* in overall rice planting has been increasing recently, particularly in south China, the main *indica* producing area of the country.

The utilization of heterosis resulted in a major breakthrough in rice breeding, and the growing popularity of hybrid rice contributed significantly to world food production (Yuan 1987). In 1970, the wild abortive cytoplasm in rice was found in Hainan province, China, and in 1976, hybrid rice cultivars were released to farmers. In 1991, the area under hybrid rice was 17.6 million hectares, i.e., 55% of the total area occupied by hybrid rice in China. In particular, the current area of hybrid *indica* cultivation comprises more than 50% of the rice planting area (Pu et al. 2015). In contrast, the development of hybrid *japonica* was very slow, as it occupied less than 3% of the total planting area of *japonica* (Deng 2008). As the development of hybrids offers a great potential to increase the yield of rice, Yuan (2000) predicted that hybrid *japonica* is the most likely to make a breakthrough in the next 30 years, which will lead to a new growth point in China’s grain production. To further facilitate the development of the hybrid *japonica* rice, we have reviewed recent progress in research on the breeding of hybrid *japonica* and analyzed existing problems, thus providing a reference for the development of hybrid *japonica* rice.

## The History of Research on Hybrid *japonica* Rice

The research on hybrid *japonica* rice was originated in Japan (Li and Wu 1991). In the 1950s, Weeraratne and Sampath first reported the phenomenon of cytoplasmic male sterility (CMS) in rice (Sampath and Mohauty, 1954). In 1958, CMS lines with the wild rice cytoplasm were obtained for the first time by Katsuo and Mizushima by backcrossing Fujisaka 5 and Chinese red-awned wild rice; however, no restorer lines were found at that time (Deng 2008). In 1966, Shinjyo and O’mura developed the first CMS lines of Baotai (BT) type in the backcross of Chinsurah Bolo II *indica* rice from India and Taichung 65 from Taiwan and found a few conspecific restorer lines, but due to the inconspicuous heterosis, the three-line varieties could not be widely promoted (Shiniyo C, O’ mura 1966; Shiniyo C 1969, 1972). Subsequently, Watanabe developed the CMS-Lead lines in the cross of Lead (Myanmar *indica*) and Fujisaka 5 (Deng 2008). American and Indian researchers developed *japonica* CMS lines with cytoplasm from Taiwan cultivars Birco and *Oryza glaberrima* (Deng 2008). The International Rice Research Institute (IRRI) launched the hybrid rice research program in 1977, and after more than 10 years of effort, they developed the first batch of hybrid rice parents and hybrid rice varieties in 1989. With the help of international organizations and China, the technology and the material were transferred to some rice-producing countries in Asia to develop the production of hybrid rice (Huang 2004).

At present, there are more than 120 rice-cultivating countries on five continents. Outside of China, 110 million ha of rice are cultivated worldwide, mostly in Asia, Africa, and the Americas (Deng 2008; Hu 2010), and the area occupied by hybrid rice reached 6.36 million ha in 2014. Of these, 5.91 million ha were in Asia, and 0.45 million ha were in South and North America. The countries growing hybrid rice included Bangladesh, Pakistan, India, Indonesia, the Philippines, Myanmar, Vietnam, Sri Lanka, Iran, United States, Brazil, Argentina and Uruguay. China, India, Bangladesh, Pakistan, Indonesia, the Philippines, Myanmar, Vietnam, and the United States are the major countries growing hybrid rice. Hybrid rice in the United States accounts for more than half of its total rice production area. However, as the *indica* hybrid was the dominant rice in these countries, the breeding and promotion of hybrid *japonica* rice were limited (Xie and Peng 2016).

## Research Progress of Hybrid *japonica* Rice in China

**Fig. 3:**
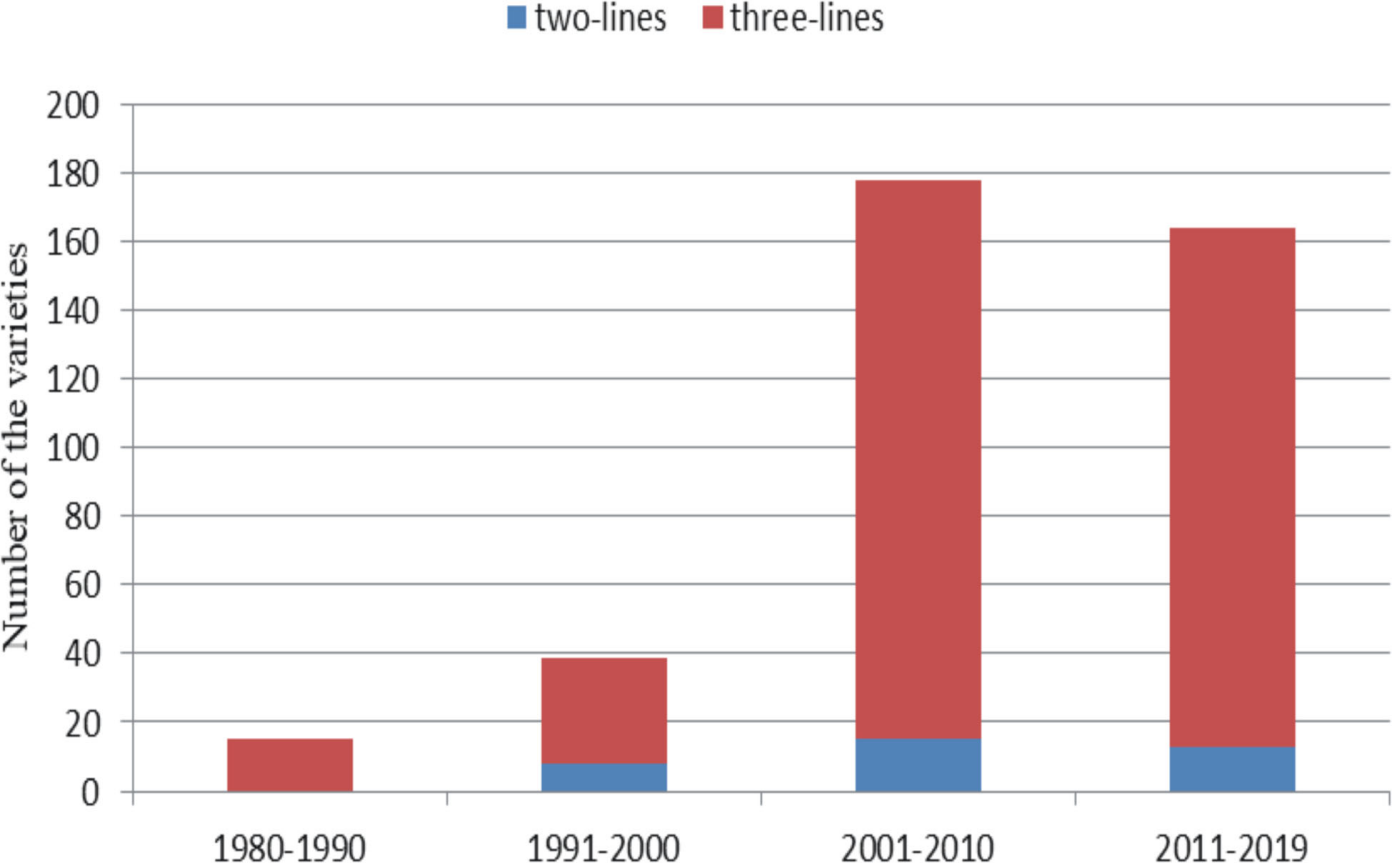
The hybrid *japonica* rice varieties bred in China

Since the 1960s, breeders led by Li Z and Yang Z have been carrying out research on hybrid *japonica* rice (Li 1977, 2000; Li and Wu JL 1991; Yang 1994, 1998, 1999, 2005, 2016). Over the past 50 years, remarkable progress has been made in the breeding of hybrid *japonica* rice. By 2019, China has approved 396 hybrid *japonica* rice combinations, whose parents, variety types, and time of being certified are listed in Table 1. As can be seen from Table 1 and Fig. 3, most of the varieties were generated between 2000 and 2019, and the hybrid *japonica* rice accounts for 88.4% of the total number of cultivars, while *indica*-*japonica* varieties for only 11.6%. Among these hybrid *japonica* rice varieties, the majority (360) are three-line varieties, and only 36 are two-line varieties.

**Table 1:** List of hybrid *japonica* rice varieties bred in China

Vareties	Female	Male	Type	Subspecies	Year of release
Li you 57	Li ming A	C57	Three-lines	Japonica	1980
Xiu you 57	Xiu ling A	C57	Three-lines	Japonica	1984
Dang you C bao	Dang xua nwan 3 A	C Bao	Three-lines	Japonica	1985
Di you 57	D 57 A	C57–10	Three-lines	Japonica	1985
Ji jing za 1	Li ming A	K55	Three-lines	Japonica	1986
Qiu you 20	Qiu guang A	F20	Three-lines	Japonica	1986
Yan you 57	Yan jing 903 A	C57V	Three-lines	Japonica	1988
Han you xiang qing	Han feng A	xiang qing	Three-lines	Japonica	1989
Liu you 3–2	Liu qian xin A	ning hui 3–2	Three-lines	Japonica	1989
Dang you 9	Dang xuan wan 2 A	wan hui 9	Three-lines	Japonica	1989
Feng you 9	Feng jin A	C79–64	Three-lines	Japonica	1989
76 you 312	76–27 A	pei C312	Three-lines	Japonica	1990
Han you 1027	Han feng A	T1027	Three-lines	Japonica	1990
Ai you 82	Dong jiu ai 4 A	hong yu 82	Three-lines	Japonica	1990
Qi you 6	76-27A	2674	Three-lines	Japonica	1990
Xun za 29	Dian xun 1 A	nan 29	Three-lines	Japonica	1991
Wan dao 16	Liu qian xin A	Cbao	Three-lines	Japonica	1992
Xin dao 4	–	–	Three-lines	Japonica	1992
Xin dao 3	Liao 10120A	hui 73–28	Three-lines	Japonica	1992
Wan dao 18	Liu qian xin A	82,022	Three-lines	Japonica	1992
Xu you 3–2	Xu dao 2 A	ning hui 3–2	Three-lines	Japonica	1993
Si you 422	Si dao 8 A	lun hui 422	Three-lines	Japonica	1993
Jing you 6	Zhong zuo 59 A	jin 1244–2	Three-lines	Japonica	1993
Wan dao 26	7001S	xiu shui 04	Two-lines	Japonica	1994
Wan dao 22	80–4 A	wan hui 9	Three-lines	Japonica	1994
70 you 9	7001S	wan hui 9	Two-lines	Japonica	1994
Ning you 1	552 A	FR-79	Three-lines	Japonica	1994
Si you 9083	Si dao 8 A	C9083	Three-lines	Japonica	1994
E jing za 1	N5088S	R187	Two-lines	Japonica	1995
8 you 161	8204 A	R161	Three-lines	Japonica	1995
Hua jing za 1	7001S	R1514	Two-lines	Japonica	1995
Yu za 29	Dian yu 1 A	nan 29–1	Three-lines	Japonica	1995
Wan dao 34	80–4 A	HP121	Three-lines	Japonica	1996
9 you 138	Xu 9201 A	N138	Three-lines	Japonica	1996
Liu you 3	Liu qian xin A	yin hui 3	Three-lines	Japonica	1996
Qi you7	76–27 A	K1457	Three-lines	Japonica	1996
Wan dao 48	7001S	shuang jiu	Two-lines	Japonica	1997
Si you 9022	Sidao 8 A	C9022	Three-lines	Japonica	1997
Qiu you 62	Qiu guang A	C9162	Three-lines	Japonica	1997
Wan dao 46	80–4 A	T1027	Three-lines	Japonica	1997
Ti you 418	Ti jin A	C418	Three-lines	Japonica	1998
Min you 128	83 A	R128	Three-lines	Japonica	1998
Liu you 121	Liu qian xin A	HP121	Three-lines	Japonica	1998
Si you 88	Sidao 8 A	hui 88	Three-lines	Japonica	1998
Si you 418	Sidao 8 A	C418	Three-lines	Japonica	1999
Wan dao 50	4008S	xiu shui 04	Two-lines	Japonica	1999
si you 523	Sidao 8 A	R523	Three-lines	Japonica	1999
jin jing za 1	LS2S	zhong zuo93	Two-lines	Japonica	1999
9 you 418	Xu 9201 A	C418	Three-lines	Japonica	2000
yong you 1	Ning 67 A	K1722	Three-lines	Japonica	2000
ning you 2	401 A	R253	Three-lines	Japonica	2000
yun guang 8	N5088S	yun hui 11	Two-lines	Japonica	2000
8 you 682	Xu 8908 A	R37682	Three-lines	Japonica	2000
8 6you 8	863 A	ning hui 8	Three-lines	Japonica	2000
3you 18	Jin 3 A	C418	Three-lines	Japonica	2001
liao you 5218	Liao 5216 A	C418	Three-lines	Japonica	2001
hua jing za 2	N5088S	41,678	Two-lines	Japonica	2001
yongyou 2	Yongjing 2 A	K1722	Three-lines	Japonica	2001
siyou 12	Sidao 8 A	Z12	Three-lines	Japonica	2001
liao you 3418	Liao 326A	C418	Three-lines	Japonica	2001
jin jing za 3	Zao hua dong A	c you 1	Three-lines	Japonica	2001
69 you 8	Xu 69A	R11238	Three-lines	Japonica	2001
yan you 1	Yan jing 5 A	yan hui 93,005	Three-lines	Japonica	2001
liao you 4418	Xiu ling A	C418	Three-lines	Japonica	2001
liao you 5	Liao yan 28 A	504–6	Three-lines	Japonica	2001
jin jing za 2	Jin 3 A	C272	Three-lines	Japonica	2001
IIIyou 98	MH2003 A	R18	Three-lines	Japonica	2002
chang you 1	Wu yun jing 7 A	shen hui 254	Three-lines	Japonica	2002
yongyou 3	Yongjing 2 A	K1863	Three-lines	Japonica	2002
dian za 31	Yu mi 15 A	nan 34	Three-lines	Japonica	2002
shen you 1	8204 A	shen hui 1	Three-lines	Japonica	2002
liu you 8	Liu qian xin A	HP121–8	Three-lines	Japonica	2002
dian za 32	Li yu A	nan 34	Three-lines	Japonica	2002
minyou 55	261S	min55	Two-lines	Japonica	2002
jin jing za 4	502 A	R411	Three-lines	Japonica	2002
liao you 1518	Liao 151 A	C418	Three-lines	Japonica	2002
yun guang 9	7001S	yun hui 124	Two-lines	Japonica	2002
liao you 0201	Liao 02 A	C01	Three-lines	Japonica	2002
pu you 801	69 A	J60	Three-lines	Japonica	2002
86you 242	863 A	R242	Three-lines	Japonica	2002
ba you 8	8204 A	R9525	Three-lines	Japonica	2002
xin za jing 1	Pei ai 64S	yu jing 3	Two-lines	Japonica	2003
jin jing za 5	Zao hua dong A	773	Three-lines	Japonica	2003
xiang you 18	Ai zhi xiang A	MR18	Three-lines	Japonica	2003
liang you pei jing	Pei ai 64S	94,205	Two-lines	Japonica	2003
ning you 3	Zhong zuo59 A	1229	Three-lines	Japonica	2003
wan dao 74	80–4 A	wan hui 98	Three-lines	Japonica	2003
yan you 2	Yan 93,538 A	lun hui 422	Three-lines	Japonica	2003
jing you 15	Zhong zuo 59 A	Y772	Three-lines	Japonica	2003
shen you 4	Shen 4 A	xiang qing	Three-lines	Japonica	2003
liao you 14	Liao 30 A	C4115	Three-lines	Japonica	2003
yun guang 12	95076S	yun hui 124	Two-lines	Japonica	2003
minyou xiang jing	261S	Wxiang 99,075	Two-lines	Japonica	2003
yan liang you 2818	GBO28S	C418	Two-lines	Japonica	2003
jin you 2003	341 A	773	Three-lines	Japonica	2003
wan dao 72	80–4 A	2277	Three-lines	Japonica	2003
yong you 4	Yong jing 2 A	K2001	Three-lines	Japonica	2003
wan dao 70	80–4 A	MR19	Three-lines	Japonica	2003
dong jing za 3	N5088S	minhui 128	Two-lines	Japonica	2004
liao you 16	Liao 30 A	C272	Three-lines	Japonica	2004
wan dao 80	Shuang jiu A	wan hui 3402	Three-lines	Japonica	2004
wan dao 78	Y A	9 M059	Three-lines	Japonica	2004
yu za 34	Dian yu 1 A	nan 34	Three-lines	Japonica	2004
wan han you 1	N422S	R8272	Two-lines	Japonica	2004
dian za 33	Yu mi 15 A	dian nong R-3	Three-lines	Japonica	2004
wan dao 76	Ai zhi xiang A	MC20518	Three-lines	Japonica	2004
jing you 14	Zhong zuo 59 A	jin dao 1229	Three-lines	Japonica	2004
shen you 254	Shen 6 A	shen hui 254	Three-lines	Japonica	2004
10 you 18	10 A	R148	Three-lines	Japonica	2004
yong you 6	Yongjing 2 A	K4806	Three-lines	Japonica	2005
xiu you 5	Xiu shui 110 A	xiu hui 69	Three-lines	Japonica	2005
jia you 1	Jia 60 A	jia hui 40	Three-lines	Japonica	2005
liao you 1052	105 A	C52	Three-lines	Japonica	2005
jia le you 2	151 A	DH32	Three-lines	Japonica	2005
liao you 853	Nong lin 150A	R853	Three-lines	Japonica	2005
chang you 3	Wu yun jing 7 A	R192	Three-lines	Japonica	2005
yong you 5	Yong nuo 2 A	K6926	Three-lines	Japonica	2005
jing you 13	Zhong zuo 59 A	lu hui 3	Three-lines	Japonica	2005
zhe you 9	5016 A	zhe hui 9816	Three-lines	Japonica	2005
shen you 693	Shen 6 A	R693	Three-lines	Japonica	2005
xu you 201	Xu 9320 A	xu hui 201	Three-lines	Japonica	2005
su you 22	Wu yun jing 7 A	R16189	Three-lines	Japonica	2005
zhong jing you 1	Jin 6 A	jin hui 1	Three-lines	Japonica	2005
chang you 2	Wu yun jing 7 A	C53	Three-lines	Japonica	2005
liao you 2006	Liao 20 A	C2106	Three-lines	Japonica	2005
yong you 8	Yong jing 3 A	K6876	Three-lines	Japonica	2006
qiu you jin feng	Qiu feng A	R44	Three-lines	Japonica	2006
ai you 39	Ai zhi xiang A	MR39	Three-lines	Japonica	2006
shen you 8	Shen 4 A	R8	Three-lines	Japonica	2006
dian za 80	Dian I-11A	nan 34	Three-lines	Japonica	2006
ling xiang you 18	Ling xiang A	YC418	Three-lines	Japonica	2006
jin jing you 68	Jin 1007A	jin hui 68	Three-lines	Japonica	2006
yongyou 1460	Yong jing 2 A	T1460	Three-lines	Japonica	2006
wan dao 88	9201 A	R-8	Three-lines	Japonica	2006
jin jing you 88	Jin 1007 A	jin hui 88	Three-lines	Japonica	2006
ba you 52	8204 A	Z052	Three-lines	Japonica	2006
liao you 2016	Liao 20 A	C216	Three-lines	Japonica	2006
liao you 2015	Liao 20 A	C4115	Three-lines	Japonica	2006
dian za 36	He xi 42–7 A	nan 36	Three-lines	Japonica	2006
jin jing you 28	Jin 1007 A	jin hui 28	Three-lines	Japonica	2006
liao you 5224	Liao 5216 A	C124	Three-lines	Japonica	2006
dian za 35	He xi 42–5 A	nan 34	Three-lines	Japonica	2006
ling feng you 18	Ling feng A	SJR218	Three-lines	Japonica	2006
xu you 631	Xu 9320A	xu hui 11,631	Three-lines	Japonica	2006
chun you 2	Chun jiang 12 A	CH89	Three-lines	Japonica	2006
su jing you 2	8006 A	xiang qing	Three-lines	Japonica	2006
jin you 2006	Jin dao 341 A	C4115	Three-lines	Japonica	2006
yong you 9	Yong jing 2 A	K6093	Three-lines	Japonica	2007
yong you 10	Yong nuo 2 A	K6962	Three-lines	Japonica	2007
jia you 2	Jia 60 A	jia hui 30	Three-lines	Japonica	2007
xu you 733	Xu 364 A	xu hui 11,733	Three-lines	Japonica	2007
dian you 34	Dian jing you 1 A	nan 34	Three-lines	Japonica	2007
chun you 58	Chun jiang 12 A	CH58	Three-lines	Japonica	2007
jing za you 1	80–4 A	jing hui 1	Three-lines	Japonica	2007
chang you 4	Wu yun jing 7 A	CR-25	Three-lines	Japonica	2007
shuang you 3404	Shuang jiu A	wan hui 3404	Three-lines	Japonica	2007
tian xie 13	Xu 9320 A	xu hui 11,733	Three-lines	Japonica	2007
shen you 9723	Shen 97 A	R4023	Three-lines	Japonica	2007
jin jing you 180	Jin 5 A	R180	Three-lines	Japonica	2007
su jing you 3	9703 A	xiang qing	Three-lines	Japonica	2007
yong you 11	Yong jing 2 A	K216211	Three-lines	Japonica	2007
liao you 5273	Liao 5216 A	C73	Three-lines	Japonica	2007
zhong jing you 470	Zhong zuo 59 A	C470	Three-lines	Japonica	2007
xiu you 169	jia hua 1 A	XR69	Three-lines	Japonica	2007
T you 5	951 A	R981	Three-lines	Japonica	2007
5 you 135	jin 5 A	C135	Three-lines	Japonica	2007
jin jing you 116	jin 6 A	R116	Three-lines	Japonica	2007
hua you 14	shen 9 A	fan 14	Three-lines	Japonica	2008
xin dao 22	LA3	LC64	Three-lines	Japonica	2008
ti you 267	ti jin A	C267	Three-lines	Japonica	2008
5 you 280	jin 5 A	R280	Three-lines	Japonica	2008
qiu you 118	qiu feng A	R118	Three-lines	Japonica	2008
5 you 190	jin 5 A	R190	Three-lines	Japonica	2008
zhong jing you 8	jin jing 12 A	jin hui 3	Three-lines	Japonica	2008
fu you 135	fu A	C135	Three-lines	Japonica	2008
xu 2you 1	xu 20,111 A	xu hui 201	Three-lines	Japonica	2008
6you 160	jin 6 A	R160	Three-lines	Japonica	2008
zhe jing you 1	zhe jing 2 A	zhe jing hui 04–02	Three-lines	Japonica	2008
zhe you 10	8204 A	zhe hui 9816	Three-lines	Japonica	2008
xin 8 you 122	xin 8 A	GR03122	Three-lines	Japonica	2008
zhe you 12	zhe 04 A	zhe hui H414	Three-lines	Japonica	2008
chun you 59	chun jiang 16 A	CH59	Three-lines	Japonica	2009
yun liang you 144	2301S	yun R144	Two-lines	Japonica	2009
jin 7you 18	jin feng 7 A	jin hui 18	Three-lines	Japonica	2009
xiu you 378	xiu shui 3 A	XR78	Three-lines	Japonica	2009
zhe jing you 2	zhe jing 3 A	zhe jing hui 04–02	Three-lines	Japonica	2009
shen you fan 15	shen 10 A	shen fan 15	Three-lines	Japonica	2009
chun you 172	chun jiang 12 A	C172	Three-lines	Japonica	2009
liao you 9573	liao 95 A	C73	Three-lines	Japonica	2009
jin 7 you 58	jin feng 7 A	jin hui 58	Three-lines	Japonica	2009
95 you 161	95,122 A	R161	Three-lines	Japonica	2009
chun you 658	chun jiang 16 A	CH58	Three-lines	Japonica	2009
dian za 86	D5 A	nan 34	Three-lines	Japonica	2009
xu you 502	xu 8908 A	xu hui 502	Three-lines	Japonica	2009
zhong jing you 13	jin jing 13 A	jin hui 3	Three-lines	Japonica	2009
yong you 14	yong jing 3 A	F5006	Three-lines	Japonica	2009
5 you 360	jin 5 A	R360	Three-lines	Japonica	2009
dian za 40	chu jing 23 A	nan 34	Three-lines	Japonica	2009
jia you 608	jia 60 A	jia hui 82	Three-lines	Japonica	2009
3 you 88	jin 3 A	LC50–88	Three-lines	Japonica	2009
xin han you 26	pei ai 64S	99,026	Two-lines	Japonica	2009
liao you 1498	14 A	C198	Three-lines	Japonica	2009
yun you 948	G2480 A	yun R948	Three-lines	Japonica	2009
dian za 501	D5 A	Y-11	Three-lines	Japonica	2009
ji liao za you 1	liao 99 A	C746	Three-lines	Japonica	2009
xin dao 25	LA3	LC109	Three-lines	Japonica	2009
jia you 3	jia 335 A	jia hui 32	Three-lines	Japonica	2009
xu 68 you 201	xu 91,068 A	xu hui 201	Three-lines	Japonica	2009
jin 9you 78	jin feng 9 A	jin hui 78	Three-lines	Japonica	2009
dian za 41	he xi 42–7 A	nan 43	Three-lines	Japonica	2009
ba you 315	8204 A	zhe hui H315	Three-lines	Japonica	2009
zhong zhong you 2005	25 A	R18	Three-lines	Japonica	2009
yong you 12	yong jing 2 A	F5032	Three-lines	Japonica	2010
yun guang 101	N95076S	yun jing hui 1	Two-lines	Japonica	2010
jia le you 100	151 A	GR100	Three-lines	Japonica	2010
su you 72	su 77 A	su hui 162	Three-lines	Japonica	2010
jin jing you 132	jin jing 13 A	jin hui 2	Three-lines	Japonica	2010
chang you 5	chang 01–11 A	CR-27	Three-lines	Japonica	2010
jing liang you 5975	0259S	gr75	Two-lines	Japonica	2010
han you 8	hu han 2 A	xiang qing	Three-lines	Japonica	2010
zhong jing you 15	jin jing 12 A	jin hui 5	Three-lines	Japonica	2010
yong you 13	yong jing 3 A	F5032	Three-lines	Japonica	2010
dian za 37	he xi 42–7 A	yin hui 1	Three-lines	Japonica	2010
liao you 9906	liao 99 A	C2106	Three-lines	Japonica	2010
yong you 7	yong jing 3 A	K6262	Three-lines	Japonica	2010
long you 1715	long 17 A	R1415	Three-lines	Japonica	2010
jia pu you 608	jia 335 A	jia hui 52	Three-lines	Japonica	2010
bi jing za 2035	BJ-1 A	ZC2035	Three-lines	Japonica	2010
shen you 1	5 A	C3	Three-lines	Japonica	2010
jing liang you 2847	NC228S	R4769	Two-lines	Japonica	2010
jia you 5	jia 335 A	jia hui 125	Three-lines	Japonica	2010
dian you 35	DHC-10 A	nan 34	Three-lines	Japonica	2010
dan jing you 8	dan jing 4 A	dan hui 8	Three-lines	Japonica	2011
dian za 46	he xi 42–7 A	nan 46	Three-lines	Japonica	2011
jing you 558	jing 139 A	R558	Three-lines	Japonica	2011
shen you 16	shen 46 A	shen fan 16	Three-lines	Japonica	2011
yun guang 109	N95076S	yun jing hui 7	Two-lines	Japonica	2011
yun guang 104	N95076S	yun jing hui 4	Two-lines	Japonica	2011
xin jing you 1	xin dao 97,200 A	xin hui 3	Three-lines	Japonica	2011
xin dao 38	LA28	LC109	Three-lines	Japonica	2011
yun guang 107	yun jing 202 s	yun jing hui 7	Two-lines	Japonica	2011
jiao za jing 1	jiao 31 A	jiao hui 2	Three-lines	Japonica	2011
dong jing you 775	dong wan 17 A	jing xiang 75	Three-lines	Japonica	2011
dian za 94	D5A	Y-16	Three-lines	Japonica	2011
yong you 17	yong jing 4 A	yonghui 12	Three-lines	Japonica	2012
zhe you 18	zhe 04 A	zhe hui 818	Three-lines	Japonica-indica	2012
dian you 38	DHC-10 A	dian nong R-5	Three-lines	Japonica	2012
chun you 618	chun jiang 16 A	C18	Three-lines	Japonica	2012
shuang you 18	shuang jiu A	C418	Three-lines	Japonica	2012
shen you C9	shen wu 1 A	shen hui C9	Three-lines	Japonica	2012
6you 53	1586S	xin jing 5003	Two-lines	Japonica	2012
bao jing za 2	N95076S	BR-4	Two-lines	Japonica	2012
xin dao 40	LRA3	LRC64	Three-lines	Japonica	2012
dian za 701	D5 A	dian kun xiang 4	Three-lines	Japonica	2012
jia he you 555	jia he 212 A	jia he hui 555	Three-lines	Japonica	2012
jin hui you 50	jin hui A	JP50	Three-lines	Japonica	2012
qin na 1	yan nong S	hun he fu ben	Two-lines	Japonica	2012
dong jing you 763	dong wan 17 A	xiang hui 63	Three-lines	Japonica	2012
dian za 49	he xi 42–7 A	nan 50	Three-lines	Japonica	2012
yongyou 16	yong jing 8 A	yonghui 12	Three-lines	Japonica	2012
jia you 6	jia 335 A	jia hui 69	Three-lines	Japonica	2012
dian you 37	DHC-10 A	dian nong R-3	Three-lines	Japonica	2012
jia chang you 7	qiao feng A	hui 135	Three-lines	Japonica	2012
yong you 538	yong jing 3 A	F7538	Three-lines	Japonica-indica	2013
chun you 84	chun jiang 16 A	C84	Three-lines	Japonica	2013
yongyou 2640	yong jing 26 A	F7540	Three-lines	Japonica-indica	2013
gang you 1	071 A	C419	Three-lines	Japonica	2013
long jing 1550	yan feng 47S	liao xing 1	Two-lines	Japonica	2013
wu you 17	wu A	C17	Three-lines	Japonica	2013
yong you 1640	yong jing 16 A	F7540	Three-lines	Japonica	2013
dian he you 34	H479A	nan 34	Three-lines	Japonica	2013
yong you 720	yong jing 7 A	yong hui 20	Three-lines	Japonica	2013
chang you jing 6	chang 119 A	CR-312	Three-lines	Japonica	2013
zhe nuo you 1	zhe nuo 1 A	zhe nuo hui 04–01	Three-lines	Japonica	2013
T12you 66	T4012 A	R7066	Three-lines	Japonica	2013
jing liang you 5519	N55s	R19	Two-lines	Japonica	2013
18 you 75	18 A	R1575	Three-lines	Japonica	2013
pu you 22	ai jing 15 S	pu hui 22	Two-lines	Japonica	2013
jin jing you 11	jin jing 11 A	jin hui 1	Three-lines	Japonica	2013
ji you 1769	T176 A	C269	Three-lines	Japonica	2013
tong you jing 1	yang fu jing 7 A	R98	Three-lines	Japonica	2013
yongyou 1540	yong jing 15 A	F7540	Three-lines	Japonica-indica	2014
zhe you 13	zhe 04 A	zhe hui H813	Three-lines	Japonica	2014
ji you 3985	639 A	ji jing 85	Three-lines	Japonica	2014
yong you 1109	yong jing 11 A	F7509	Three-lines	Japonica	2014
shen you 17	shen wu 1 A	shen fan 17	Three-lines	Japonica	2014
IIIyou 304	2003 A	XH04	Three-lines	Japonica	2014
long you 467	long 3 A	R467	Three-lines	Japonica	2014
dan jing you 1	dan jing 4 A	dan hui 1	Three-lines	Japonica	2014
jing you 106	jing 139 A	C2106	Three-lines	Japonica	2014
re jing you 35	re jing 1A	jing hui 35	Three-lines	Japonica	2014
jiao yuan you 69	jiao yuan 5A	JP69	Three-lines	Japonica	2014
dian kun you 8	K5 A	S8	Three-lines	Japonica	2014
yong you 362	yong jing 5 A	F7562	Three-lines	Japonica	2014
chun you 149	chun jiang 19 A	CH149	Three-lines	Japonica	2014
liao 16you 06	liao 5216 A	C2106	Three-lines	Japonica	2014
liao 73you 62	liao 73 A	C62	Three-lines	Japonica	2014
yong you 1538	yong jing 15 A	F7538	Three-lines	Japonica-indica	2015
shen you 24	shen 01 A	shen fan 24	Three-lines	Japonica	2015
dian he you 56	yu mi 15 A	nan 56	Three-lines	Japonica	2015
5 you 68	5 A	R68	Three-lines	Japonica	2015
dong nuo you 91	dong nuo 19 A	nuo hui 11	Three-lines	Japonica	2015
yong you 4949	yong jing 49 A	F9249	Three-lines	Japonica-indica	2015
76 liang you 5	95076S	bao hui 5	Two-lines	Japonica	2015
qiu you 336	qiu 15 A	R336	Three-lines	Japonica	2015
dian he you 4106	he xi 42–7 A	yin hui 106	Three-lines	Japonica	2015
yong you 1140	yong jing 6 A	F7540	Three-lines	Japonica-indica	2015
tian long you 619	L6 A	R19	Three-lines	Japonica	2015
yong you 7850	yong jing 78 A	F9250	Three-lines	Japonica-indica	2015
dian he you 55	yu mi 15 A	nan 55	Three-lines	Japonica	2015
jing you 586	jing 139 A	C586	Three-lines	Japonica	2015
yong you 4350	yong jing 43 A	F9250	Three-lines	Japonica-indica	2015
bi jing you 210	bi jing 2 A	NR210	Three-lines	Japonica	2015
yong you 4550	yong jing 45 A	F9250	Three-lines	Japonica-indica	2015
yong you 150	yong jing 2 A	F9250	Three-lines	Japonica-indica	2016
bi jing you 3	bi jing 2A	bi jing hui 3	Three-lines	Japonica	2016
yong you 4901	A49	F8001	Three-lines	Japonica-indica	2016
jia you zhong ke 3	jia 66 A	zhong ke jia hui 1293	Three-lines	Japonica-indica	2016
zhe you 21	zhe 04 A	zhe hui F1121	Three-lines	Japonica	2016
dian he you 6612	yu mi 15 A	nan 6612	Three-lines	Japonica	2016
jiao yuan you 1	jiao yuan 3 A	jiao hui 1	Three-lines	Japonica	2016
yongyou 4149	yong jing 41 A	F9249	Three-lines	Japonica-indica	2016
yongyou 4912	yong jing 49 A	F7512	Three-lines	Japonica-indica	2016
pu you 201	pu jing 06 A	T201	Three-lines	Japonica-indica	2016
jia he you 1	jia he 212 A	hui SC01–1	Three-lines	Japonica-indica	2016
yong you 540	yong jing 3 A	F7540	Three-lines	Japonica-indica	2016
yong you 7050	A70	F9250	Three-lines	Japonica-indica	2016
yong you 8050	yong jing 80 A	F9250	Three-lines	Japonica-indica	2016
jia you zhong ke 1	jia 66 A	zhong ke jia hui 1	Three-lines	Japonica-indica	2016
zi xiang you 24	zi xiang A	shen fan 24	Three-lines	Japonica	2016
zhe you 19	zhe 04 A	zhe hui F1015	Three-lines	Japonica-indica	2016
dian he you 6611	yu mi 15 A	nan 6611	Three-lines	Japonica	2016
yong you 4953	yong jing 49 A	F6853	Three-lines	Japonica-indica	2017
zhong jia you 6	jia he 316 A	zhong hui 7206	Three-lines	Japonica-indica	2017
jin jing you 2018	jin 20 A	jin hui 18	Three-lines	Japonica	2017
jia he you 7245	jia he 212 A	zhong hui 7245	Three-lines	Japonica	2017
chun you 115	chun jiang 16 A	CH115	Three-lines	Japonica-indica	2017
shen you 26	shen 9 A	shen hui 26	Three-lines	Japonica	2017
qian jing you 57	163 A	NR210	Three-lines	Japonica	2017
jing you 165	jing 139 A	C165	Three-lines	Japonica	2017
jia you zhong ke 6	jia 66 A	zhong ke 6	Three-lines	Japonica	2017
qiu you 122	qiu 9 A	R122	Three-lines	Japonica	2017
chang you 2	chang jing 1 A	hui KF2	Three-lines	Japonica-indica	2017
chang you 312	chang 132 A	CR-312	Three-lines	Japonica	2017
yong you 7860	yong jing 78 A	F6860	Three-lines	Japonica-indica	2017
yong you 5552	yong jing 55 A	F6852	Three-lines	Japonica-indica	2017
chun you 984	chun jiang 99 A	C84	Three-lines	Japonica-indica	2017
tian long you 518	long 5 A	C818	Three-lines	Japonica	2017
jin jing you 1918	jin 19 A	jin hui 18	Three-lines	Japonica	2017
yong you 4543	yong jing 45 A	F7543	Three-lines	Japonica-indica	2017
shen you 415	shen 9 A	C415	Three-lines	Japonica	2017
lian 8 you 3	lian 8 A	yun R3	Three-lines	Japonica	2017
zhe jing you 1578	zhe jing 7 A	zhe hui 1578	Three-lines	Japonica	2017
yong you 7861	yong jing 78 A	F6861	Three-lines	Japonica-indica	2017
yong you 1662	yong jing 16 A	F6862	Three-lines	Japonica-indica	2017
jing you 1	jing 1 A	guang hui 1	Three-lines	Japonica	2017
hua zhong you 1	hua zhong 1 A	hui 16	Three-lines	Japonica-indica	2017
jiao yuan you 5	jiao yuan 3 A	jiao hui 5	Three-lines	Japonica	2017
yong you 5550	yong jing 55 A	F9250	Three-lines	Japonica-indica	2017
7you 1	7 A	yun R1	Three-lines	Japonica	2017
chun you 927	chun jiang 16 A	C927	Three-lines	Japonica-indica	2017
jiao yuan you 6	jiao yuan 2 A	jiao hui 6	Three-lines	Japonica-indica	2018
chun you 284	chun jiang 23 A	C84	Three-lines	Japonica	2018
shen you 114	shen 01 A	C14	Three-lines	Japonica-indica	2018
yong you 7753	yong jing 77 A	F6853	Three-lines	Japonica-indica	2018
xiu you 7113	xiu 71 A	XR13	Three-lines	Japonica-indica	2018
yong you 6760	yong jing 67 A	F6860	Three-lines	Japonica-indica	2018
jiang liang you 7901	jiang 79S	jiang hui 1501	Two-lines	Japonica	2018
qiu you 23	qiu 23 A	R23	Three-lines	Japonica	2018
qian jing you 2	163 A	qian jing hui 2	Three-lines	Japonica	2018
shen wu you 26	shen wu 1A	shen hui 26	Three-lines	Japonica	2018
shen 9you 09	shen 9 A	shen hui 9	Three-lines	Japonica	2018
zhong he you 1	jia he 212 A	NP001	Three-lines	Japonica	2018
jing you 653	jing 65 A	C315	Three-lines	Japonica	2018
xiu you 207	xiu shui 134 A	R207	Three-lines	Japonica	2018
zi xiang you 26	zi xiang A	shen hui 26	Three-lines	Japonica	2018
bi jing you 5	67 A	NR210	Three-lines	Japonica	2018
shu you 9	jia 81 A	zhong ke jia hui 1308	Three-lines	Japonica	2019
pu jing you 701	pu jing 06 A	PR701	Three-lines	Japonica	2019
chun you 584	chun jiang 25 A	C84	Three-lines	Japonica	2019
chang you 998	chang 386 A	CR998	Three-lines	Japonica	2019
yong you 6711	yong jing 67 A	F5711	Three-lines	Japonica-indica	2019
jin liang you 852	jin rui 8S	yun hui 503	Two-lines	Japonica	2019
yong you 1526	yong jing 15 A	F4926	Three-lines	Japonica-indica	2019
shen you 27	shen 10 A	shen hui 26	Three-lines	Japonica	2019
zhe jing you 6153	zhe jing 7 A	zhe jing hui 6153	Three-lines	Japonica	2019
yong you 7053	yong jing 70 A	F6853	Three-lines	Japonica-indica	2019
yong you 6763	yong jing 67 A	F6863	Three-lines	Japonica-indica	2019
yun liang you 504	yun jing 208S	yun hui 504	Two-lines	Japonica	2019
pu jing you 201	pu jing 06 A	PR201	Three-lines	Japonica	2019
liao 99you 30	liao 99 A	C30	Three-lines	Japonica	2019
jia you 8	jia 74 A	jia hui 8	Three-lines	Japonica	2019
yong you 5526	yong jing 55 A	F4926	Three-lines	Japonica-indica	2019
chang you jing 7	chang 410–2 A	CR-928	Three-lines	Japonica	2019
liao 99you 15	liao 99 A	C415	Three-lines	Japonica	2019
yong you 5518	yong jing 55 A	F4918	Three-lines	Japonica-indica	2019

### A Three-Line System for Hybrid Japonica Rice

The three-line system is composed of sterile line, maintainer line, and restorer line. The discovery of sterile lines enabled large-scale production of hybrid rice. The key to the breeding of hybrid japonica rice is finding suitable restorer lines that can restore the male sterile lines and improve the heterosis.

#### Dian-Type Male Sterile Lines

The study of hybrid *japonica* rice in China began in 1965. A naturally sterile plant was found in the Taipei 8, and was used as a female to develop the CMS-Dian I via nuclear replacement from *japonica* cultivar Hongmaoying in 1969. CMS-Dian I was the first and the most significant *japonica* CMS line in China (Li 2000; Yang 2016). Subsequently, *japonica* CMS lines, such as Dian-II, Dian-III, Dian-IV, Dian-V, Dian-VI, Dian-VII, and Dian-VIII were generated in the same manner. Although the lack of restorer lines limited the application of these CMS-Dian lines in the three-line system, they formed the foundation for the development of *japonica* hybrid rice in southwest, northwest, and eastern provinces of China (Li 1977). With the breakthrough achievement of creating Dian-type hybrid *japonica* rice, a number of stable CMS lines (Dianyu 1A, Dianxun 1A, Liyu A, and Yumi 15A) were developed, which were further used to create other hybrid *japonica* varieties, such as Yuza 29, Xunza 36, Dianza 32, Dianza 31, Yunguang 8, Yunguang 9, Yunguang 12, and Yunguang 14. These varieties not only exhibit a higher production of hybrid seeds but also display stronger heterosis and higher resistance to the blast disease in the F_1_ generation (Huang 2004; Yang 2016).

#### BT-Type Male Sterile Lines

In 1972, the CMS-BT line Taichung 65 was introduced from Japan by the Liaoning Academy of Agricultural Sciences (LAAS) and the Chinese Academy of Agricultural Sciences. This line was used as the female parent to develop several CMS-BT lines, such as Liming A, Xiuling A, Akihikari A, Sasanishiki A, Liuqiangxin A, Sidao 8A, Wuyunjing 7A, Hanfeng A, Xiushui 4A, Zhong 7941A, 41A, Ning 67, and Yongjing 2A. However, all these lines lacked appropriate *japonica* restorer lines (Deng 2008). In 1975, using the “*indica-japonica* bridging technique”, LAAS introduced restorer genes from *indica* to *japonica* by backcross of IR8 / Keqing 3 // Keqing 3. As a result, the *japonica* restorer line C57 with strong combining ability was developed, and the first hybrid *japonica* rice variety, Liyou 57, was successfully bred and was widely cultivated in China. This progress greatly promoted the research on hybrid *japonica* rice and its and utilization in northern China (Yang 1994). Many *japonica* restorer lines and their combinations were developed from C57 and its offspring for application in Beijing, Anhui, Zhejiang, Jiangsu, Hebei, Tianjin, and other provinces of China. This achievement resulted in a remarkable increase in the yield per unit area in northern China (Yang 2005). After the creation of the C57 line, the *japonica* restorer line C418 was developed by introducing wide-compatible genes, allowing to generate a series of hybrid rice combinations, such as Tiyou 418, Siyou 418, 9 You 418, and 3 You 18, and further promoting the development of hybrid rice (Yang 1998, 1999). The introduction of super-hybrid rice breeding project in 1998 drove continuous development in super-hybrid *japonica* rice research (Hua et al. 2002, 2006). The seed production of *japonica* hybrid rice has been a difficult problem due to the delayed flowering time and low stigma exposure of *japonica* CMS lines. LAAS and National *Japonica* Research Center (NJRC) improved genetically the stigma exposure rate in sterile lines by introducing *indica* genes responsible for the high rate of stigma exertion. Using this strategy, a number of *japonica* CMS lines with high stigma exposure rates have been bred, which include Liao 105A, Liao 30A, Liao 02A, Liao 5216A, Liao 99A, Liao 60A, Liao 166A, Liao 11A, Liao 73A, and Liao 143A (Li 1987; Ling 1989; Shen et al. 1994; Wang et al. 2008). The National *Japonica* Engineering Technology Center (NJETC), China Rice Research Institute, and Jiangsu Academy of Agricultural Sciences have also developed *japonica* CMS lines with high stigma exposure rates, such as L6A, 18A, and Chunjiang 99A (Chen et al. 2011a; Dong et al. 2016). The genetic improvement of *japonica* CMS lines increased the stigma exposure rate from less than 30% to more than 60%, reaching as high as 80% in some CMS lines (Wang et al. 2008). Further, the seed setting rate in natural outcrossings reached 40–60%, and the seed production yield was markedly increased, from 1.5–2 t/ha to 3.5–4.5 t/ha. Additionally, the flowering time of sterile lines started 30–60 min earlier, and the difference of flowering time between the parents shortened, so the problem of low seed production in hybrid *japonica* rice was solved (Wang et al. 2008). After the development of C418, a series of restorer lines with high quality, wide-compatibility, and good combining ability were created by crossing early maturity *japonica* with ideotype in north China and tropical *japonica* in Southeast Asia. Examples of these lines are C2106, C787, C419, C315, C62, C224, C397, C4115, C52, C272, C73, C238, C415, R19, and LR27. These restorer parents with tropical *japonica* background are characterized not only by wide restoring spectrum but also strong restoring ability. These restorer parents exhibit several features of the ideal plant type, such as compact plant architecture, short flag leaf, and semi-dense or dense panicle type. In addition, quality, resistance, and maturity were also improved (Zhang et al. 1999). A series of high-quality super-hybrid *japonica* varieties developed using the newly developed CMS and restorer lines were released. For instance, the hybrid *japonica* varieties include the Liaoyou 5218, Liaoyou 1518, Liaoyou 16, Liaoyou 14, Liaoyou 5273, Liaoyou 0201, Liaoyou 1052, Liaoyou 9573, Liaoyou 1498, Liaoyou 20, Liaoyou 2006, Liaoyou 2015, Liaoyou 2106, Liaoyou 5206, Liaoyou 9906, Liao99you15, Liao99you27, Liao99you30, Liao16you06, Liao73you62, Jingyou558, Jingyou106, Jingyou165 and Jingyou653 released by LAAS (Wang 2008), Jinjingza 2 and Jinjingza 4 released by the Tianjin Academy of Agricultural Science, Gangyou 1 released by the Donggang Farm in Liaoning province, and the high-quality fragrant hybrid *japonica* rice Tianlongyou 619 released by the National *japonica* rice Engineering Technology Center (NJETC). In addition, a series of hybrid *japonica* rice, such as Yongyou 3, Yongyou 538, Yongyou 12, Yongyou 2640, Chunyou 84, Chunyou 927, Changyou 1, and Shenyou 1 were also released in the southern rice-growing regions. These new hybrid *japonica* varieties have significant advantages in grain number per panicle, yield potential, quality, plant architecture, and resistance to blast, rice bacterial blight, and rice stripe disease (Dong et al. 2016).

#### WA -Type and Yinshui-Type Male Sterile Lines

In the 1970s, several research institutes in China bred WA-type *japonica* using the three-line system (Yang and Zhu 2009). However, large-scale breeding and cultivating WA-type hybrid *japonica* rice was once considered impossible due to the unavailability of restorer lines. Nevertheless, some progress has been made in recent years, such as breeding *japonica* CMS lines Nonghu 26A and Zhen 5A, and identifying corresponding restorer lines (Zhang et al. 2003). However, the WA-type CMS lines were seldom used in agriculture because of their poor restorability, poor flowering, and pollination characteristics.

In recent years, the Chinese Rice Research Institute and LAAS have studied the Yinshui-type hybrid *japonica* rice, and developed three-line varieties. Importantly, heterosis and seed production technology of Yinshui-type hybrid *japonica* continues to be investigated.

### The Two-Line System for Hybrid Japonica Rice

Studies on breeding the two-line system of hybrid *japonica* rice in China began in 1973 (Shi 1981). In 1985, the first *japonica* photo-sensitive genic male sterile (PGMS) line was generated (Luo et al. 1992). Since then, more than 20 years of research by Chinese scientists produced significant improvements in the two-line system of hybrid rice. Nuclear sterile genes of *japonica* lines currently available in China are mostly derived from Nongken 58S. The two-line system of hybrid *japonica* rice was developed rapidly in both Taihu and Yangtze valleys. Since the creation of the *japonica* male sterile line N5088S (used for both sterile and maintainer lines) in Hubei Province in the late 1990s, a series of similar lines, such as 7001S, has been developed and is now used for breeding. Trials and subsequent production in the Yangtze valley demonstrated that these derived cultivars have the advantages of high yield, high quality, and multi-resistance (Wang et al. 1994; Wang et al. 1995). Recently, the Northern Hybrid *Japonica* Research Center successfully overcame the problem of low seed setting rate of subspecies hybrids by using the two-line system. During the Chinese “Tenth Five-Year-Plan”, the Anhui Academy of Agricultural Sciences used the BT-type CMS lines as the cytoplasm donor, and the *japonica* PGMS lines as the male maintainer to create a new SA-type sterile line. This approach not only eliminated self-fertilization of BT-type CMS lines under high-temperature conditions but also effectively prevented self-fertilization of PGMS lines under low-temperature conditions (Li et al. 1997; Mou T 2016; Wang et al. 2005; Yang et al. 2008).

In northern China, GB028S was the first reported *japonica* PGMS line. It had the starting temperature for fertility conversion of approximately 22 °C (Li 1997). During the mid-1990s, *japonica* PGMS line 108S was developed to be used as sterile and maintainer lines in northern China (Wei et al. 2000).

*Japonica* photo-thermo-sensitive genic male sterile (PTGMS) lines were developed rapidly in the Yangtze valleys. For example, the N5088S, 31111S, and 31301S were generated in Hubei Province (Dong et al. 2016), Peiai 64S in Jiangsu Province (Yuan et al. 2012), 7001S, 2304S, 8087S, and 3502S in Anhui Province (Li et al. 1994; Wang et al. 2012) These PTGMS lines were used in the two-line system breeding to create a number of hybrid *japonica* varieties, such as 70You9, 70You4, 70Youshuangjiu, Ejingza 1, Ejingza 2, Liangyou 8828, Liangyou 122, Liangyou 276, Liangyouxinjing 1, and Liangyouxinjing 2 (Yang et al. 2009a, 2009b). The new varieties of Peiai 64S/C8420 and Peiai 64S/C418, obtained by crossing the *indica* PTGMS lines and *japonica* restorer lines with wide-compatibility genes, exhibited great potential for increasing the yield in northern China (Si et al. 2011).

### Progress in Genetic Studies on Traits Related to Heterosis Utilization

#### Yield and Hybrid Vigor

Increasing grain yield is a long-term goal in rice breeding dictated by the need to meet the demand for global food security. Heterosis, i.e., higher performance for a trait in the hybrid than in both parents, offers an important strategy for rice breeding. Over the years, numerous studies have focused on the biological basis of heterosis in hybrid *indica* rice. It is generally believed that yield heterosis is mediated by many mechanisms, such as genetic distance (Saghai et al. 1997; Xiao et al. 1996), dominant complementary (Xiao et al. 1995), additive by additive epistatic effects (Zhuang et al. 2002), overdominance and pseudo-overdominance (Zhou et al. 2012), allele-specific expression (Lin et al. 2019), and accumulation of excellent alleles (Huang et al. 2015). Gene mapping of yield and yield-related heterosis parameters was performed in various populations, and hundreds of heterotic agronomical traits quantitative trait loci have been mapped on almost all rice chromosomes. Many QTLs contribute to heterosis, with some exhibiting strong heterotic effects on essential agronomical traits such as grain yield, flowering time, panicle grain number, seed setting rate, growth period, and photosynthetic efficiency (Chen et al. 2010; Huang et al. 2015; Huang et al. 2016; Li et al. 2016; Xin et al. 2014). Thus far, significant progress has been made in breeding hybrid *japonica* varieties. However, the yield of hybrid *japonica* has been low and was unstable in comparison with the hybrid *indica*. To improve hybrid vigor and combining ability (CA) between hybrid *japonica* parents, 81 hybrids were created, and the CA of 18 hybrids *japonica* parents was calculated. Associated loci residing on chromosomes 2, 5, 7, 9, and 11 that recorded maximum positive values for the CA of traits were identified. It was concluded that the strategy to improve the heterosis of hybrid *japonica* rice involved pyramiding favorable SNP loci of CA and eliminating the unfavorable loci from parental genomes (Zaid et al. 2017).

#### Flowering Time

The yield of seed production in hybrid *japonica* is affected by several factors, such as physiological characteristics of parents, the technology of chemical control, cultivation methods, climate, and flowering time. For sterile lines characterized by low stigma exposure, the synchronization of flowering time with restorer lines is critical for seed production (Tong et al. 2002). The genetic factors that influence flowering time include, among others, the characteristics of the flower organ, the difference of variety type, the length and width of grain. The flowering speed of sterile lines is slower, while the flowering time occurs later than in restorer lines. Additionally, a difference in the flowering time exists between the *indica* and *japonica* varieties (Zhang et al. 2016). Typically, *indica* rice blooms early, reaching full flowering at about 11:00 am, while *japonica* rice blooms later, reaching full flowering at about 12:30 pm. Grain properties are closely related to the flowering characteristics of rice. The rounder the grain, the later it blossoms, and the longer the grain, the earlier it blossoms (Zhang et al. 2016). Due to the complexity of factors affecting the time of flowering, the research on its genetic mechanism began relatively late. Since 2010, different groups have been used to map QTLs for flower time. Using the Chuanxiang 29B/Lemont reconstituted inbred line population, three early flowering QTLs were mapped, two of them located on chromosome 10, and one on chromosome 5. The contribution rate of a major QTL reached 73.72% (Zhang et al. 2016). Another RIL population derived from Qishanzhan/Qiuguang was used in the studies on the genetic control of flowering time. The blooming time was found to be a quantitative trait controlled by multiple genes. Six QTLs located on chromosomes 1, 2, 7, 8, 10, and 12 were identified, with a contribution rate of 7.08% to 26.95% (Ma et al. 2011). Using the F_2_ population derived from WAB368-B-2-H2-HB (9:31–10:00)/Liuqianxin (11:01–11:30), 4 QTLs were mapped to chromosomes 1, 1, 10, and 12, respectively, and the contribution rate of each QTL ranged from 5.8% to 11.3% (Wan et al. 2013). Recently, the CRISPR/Cas9 technique has been introduced to breed early flowering rice lines. By editing the grain length gene GS3, long-grain *japonica* rice was created, which had an earlier flowering time than the short-grain *japonica* rice. In addition, when the flowering time of the sterile line and restorer line do not coincide, hormone spraying can also provide satisfactory results. It has been demonstrated that a methyl jasmonate (MeJA) spray effectively promotes rice flowering, and *indica* rice is more sensitive than *japonica* to the treatment with MeJA, and the response of *indica* rice is faster than that of *japonica* rice. High concentration (4 mmol/L) of MeJA applied late in the afternoon, at 5:00 pm, is the optimal treatment to promote the flowering of *japonica* rice (Zhang et al. 2016).

#### Stigma Exposure Rate

The degree of stigma exsertion is an important feature determining the seed setting rate. Increasing the seed setting rate in male sterile lines is important to achieve a high yield of hybrid seeds, and the stigma exposure rate is the main factor affecting this parameter (Dang et al. 2016). The stigma exsertion rate correlates positively with the yield of seeds; the seed setting rate in sterile lines increases by 0.74–0.92 percentage points for every 1% increase in stigma exposure rate, equivalent to an increase of at least 47 to 68 kg per hectare (Yang 2016). Stigma exposure in rice is generally considered to be a quantitative trait controlled by multiple genes, with dominant inheritance, lower epistasis effect, and greater environmental influence (Li et al. 2014a, 2014b; Ma et al. 2018). At present, nearly 60 QTLs related to stigma exposure rate have been identified (Li et al. 2014a, 2014b; Ma et al. 2018; Miyata et al. 2007; Yan et al. 2009), and are distributed throughout the 12 chromosomes of rice. Liu et al. (2015) mapped a main gene controlling the length of stigma in rice, *qSTL3*, to the 19.8 kb interval in the center of the short arm of chromosome 3 and verified the gene function. Using 227 rice germplasm as the material in the genome-wide association study, Dang et al. (2016) detected 6 QTLs regulating stigma length. Most of the stigma exsertion genes were derived from wild rice and *indica* subspecies, and prevalently had a low contribution rate, small additive effect, and were sensitive to the environmental conditions. In addition, the stigma exertion rate was higher in varieties with longer spikelets and longer stigma. Grain length, grain aspect ratio, stigma span, and ovary length were all positively correlated with stigma exposure rate.

#### CMS and Fertility Restoration

CMS, a maternally inherited inability to produce functional pollen, has been observed in more than 200 species of higher plants, and this defect is dependent on cytoplasmic genes (Hu et al. 2012). Recent studies have shown male sterility caused by CMS genes are genetically bound to mitochondria. Accordingly, a protein that restores pollen sterility is encoded by nuclear genes, known as fertility restorer genes (*Rf* genes) (Budar et al. 2003). Thus far, five rice CMS genes and eight Rf genes have been cloned (Table 2 and Table 3). As indicated in Table 2, all rice CMS genes are derived by the recombination process of the mitochondrial genome and are frequently coupled with the functional genes of mitochondrial respiratory chain to form a co-transcribed transcript of infertility genes (Liu et al. 2018). Among the five types of sterile lines, BT-CMS is the one most widely used in hybrid *japonica* rice. The BT-CMS gene *orf79* was discovered in 1994 using Southern hybridization during the analysis of mitochondrial gene recombination events. The *orf79* gene is located downstream of the mitochondrial *Atp6* gene and encodes a protein containing 79 amino acids (Akagi et al. 2004), and its effect on fertility can be reversed by restorer lines carrying the *Rf1a* and *Rf1b* genes. *Rf1* is the first known restorer gene for gametophytic male sterility. The BT-CMS restorer genes *Rf1a* and *Rf1b* encode pentatricopeptide (PPR) proteins with the length of 791 and 506 amino acids, respectively. Rf1a and Rf1b contain, respectively, 18 and 11 PPR domains, and are candidate proteins for targeting mitochondria (Wang et al. 2006).

**Table 2:** CMS types and related genes in rice

Type	Related gene	Protein characteristics	Reference
BT-CMS (G)	B-*atp6-orf79*	Membrane protein	Akag 2004
HL-CMS (G)	*atp6-orfH79*	Membrane protein	Peng et al. 2010
WA-CMS (S)	*rpl5-WA352*	Membrane protein	Luo et al. 2013
LD-CMS (G)	L-*atp6-orf79*	–	Etsuko et al. 2011
CW-CMS (G)	*orf307*	–	Fujii and Toriyama 20

**Table 3:** Restorers of fertility for CMS in rice

Type	Related genes	Protein characteristics	Reference
BT-CMS (G)	*Rf1a, Rf1b*	Triangular pentapeptide repeat structural protein (PPR)	Wang et al. 2006
HL-CMS (G)	*Rf5, Rf6*	Triangular pentapeptide repeat structural protein (PPR)	Hu et al. 2012
WA-CMS (S)	*Rf3, Rf4*	Triangular pentapeptide repeat structural protein (PPR)	Cai et al. 2012
LD-CMS (G)	*Rf2*	glycine-rich protein	Etsuko et al. 2011
CW-CMS (G)	*Rf17*	Acyl transporter synthetase	Fujii and Toriyama 2009

### Breeding Strategies

#### High Yield Breeding

The utilization of interspecific heterosis is an effective strategy to obtain super-high yield hybrid *japonica* rice. In the 1960s, strong heterosis was reported in interspecies hybrids of *indica*-*japonica*, manifested mostly by tall plant height, large panicle size, large grain number, strong tillering power, strong stem strength, highly developed root system, and strong disease resistance (Yang et al. 1962). In the 1980s, breeding of *indica*-*japonica* interspecific hybrid combinations was conducted in China, resulting in the development of certain hybrid combinations, e.g., chengte232/erliuzhaizao and 3037/02428, which increased the yield by more than 20% when compared with *indica* rice combination Xian you 63 in the same period (Deng 2008). In recent years, a series of new high-yield *indica*-*japonica* hybrid rice combinations, such as Yongyou and Chunyou, have been bred by combining sterile lines of *japonica* with *indica* restorer lines. Ningbo seed co. LTD developed Yongyou 538, which has high and stable yield. In the provincial production test, the average yield of Yongyou 538 was 11.3 t/ha, 29.6% higher than the control. Chunyou 84 was obtained by crossing the *japonica* sterile line Chunjiang 16A and *indica*-*japonica* restorer line C84; it provided an average yield of 10.3 t/ha, 22.9% higher than the control. Nevertheless, due to significant genetic differences between the parent lines, the hybrid varieties often exhibit certain undesirable traits, such as overhigh plant (Dai et al. 1991), low seed setting rate (Zhu and Liao 1990), long growth period (Yuan 2002), and poor grain filling degree (Yuan 2002). These problems can be solved to some extent by the introduction of a wide-compatibility gene or the aggregation of the specific compatibility gene S_5_^i^ in *japonica* rice **(**Chen et al. 2011b; Mi et al. 2016; Shahid et al. 2013; Wan 2010**).** In northern China, in addition to using the heterosis of *indica*-*japonica*, the emphasis is placed on the introduction of the *dep1* gene in the cultivation of many hybrid *japonica* rice varieties with upright and semi-upright panicles, such as Liaoyou 9906 and Gangyou 1. These varieties have a higher yield, denser grain, and are less lodging than the loose panicle hybrid *japonica* rice (Gao et al. 2012).

#### Quality

Hybrid rice is produced by cross-breeding of two parents with different genotypes, the genetic mechanism of quality is very complex. It was repeatedly demonstrated that the phenotypes of the majority of quality traits in hybrid F_1_ tend to be of the middle parent type (Deng 2008). Therefore, when breeding high-quality *japonica* combinations, the selection of quality traits of both parents is essential. Choosing good quality traits of both parents can effectively prevent negative separation of hybrid rice quality (Yang 2016). For example, Jingyou 653, developed by the Liaoning Rice Research Institute, is a high-quality hybrid *japonica* rice variety, whose parent 65A and C315 are both high-quality lines. The Jingyou 653 hybrid won the first prize in the Japan-China rice tasting contest activity held in Japan in 2016.

#### Disease Resistance

Rice blast is a major disease in Zhejiang, Shanghai, Tianjin, Liaoning, and other regions with large planting areas of hybrid *japonica* rice, and has a great negative impact on rice production. Prolonged rainfall during the heading stage of rice results in the frequent occurrence of rice blast causes serious losses in rice production. The most economical and effective method to control this disease is the breeding and cultivation of blast-resistant rice varieties. To date, 36 rice blast resistance genes have been cloned. Thirty-four of these genes are dominant (Yang et al. 2019), enabling the improvement of the disease resistance of rice by gene pyramiding. The hybrid combination with strong disease resistance and broad resistance spectrum can be created by breeding and aggregating multiple *japonica* hybrid parents resistant to rice blast or by combining sterile lines and restorer lines with different genes. By this approach, a large number of hybrid *japonica* rice varieties with strong resistance and broad resistance spectrum were obtained (Chu et al. 2018). For example, the hybrid *japonica* rice varieties Liao73You62 carrying the *Pid2* and *Pid3* genes exhibited resistance to rice blast in Liaoning province for many years. Additionally, the genes of resistance to bacterial blight and rice blast were combined in the same hybrid to enhance broad resistance to various diseases (Abhilash et al. 2016; Dash et al. 2016).

#### Combination Strategies

The hybrid combination model plays a particularly important role in the breeding of hybrid *japonica* rice. During many years of breeding, the combination model of *indica*-*japonica* complementation, tiller angle complementation, yield components, morphological structure complementation, and photo/temperature complementation, has been gradually developed in China (Huang et al. 2016; Yang 2016; Yu et al. 2008). However, differences in the adopted strategies continue to be present among different regions. In the southern rice-growing regions, such as the middle and lower reaches of the Yangtze River, breeders take advantage mostly of the *indica*-*japonica* hybrid, introducing the wide-compatibility genes, and selecting the hybrid varieties with big panicles, such as the Yongyou and Chunyou series. In the Huang-Huai region in central China, breeders rely mostly on the combination of photosensitive *japonica* male sterile lines and temperature-sensitive restorer lines to breed two-line hybrid *japonica* rice varieties. In the northeast rice-cultivating region, where the temperature is lower and the frost-free period is shorter, the hybrid *japonica* hybrid rice combinations with medium panicle grain number, more tillers, higher seed setting rate, and good synchronicity of filling are generally hybridized with multi-branched sterile lines and rice restorer lines with large panicles (Leng and Wang 2019; Yang 2016; Wang et al. 2011).

## Cultivation of Hybrid *japonica* Rice in China

**Fig. 4:**
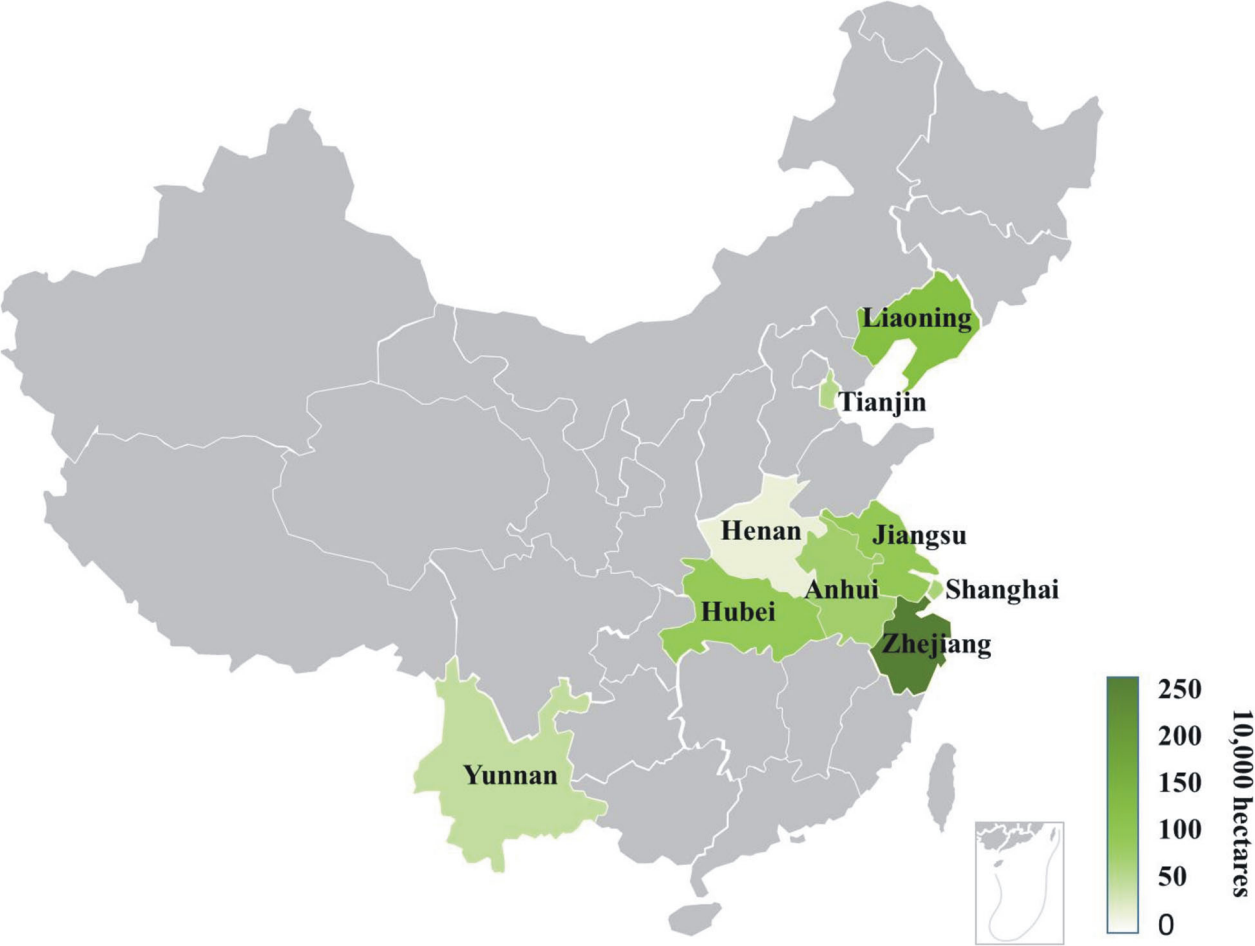
Production map of hybrid *japonica* rice of China. The provinces marked green are those with a cumulative promotion area of more than 10,000 ha from 1981 to 2018. The deeper the green degree, the larger the cumulative promotion area

Since the approval of the first *japonica* hybrid rice Liyou 57 in 1980, 396 varieties have been approved until 2019 (Table 1). Although the number of approved varieties is large, only some of them can be widely cultivated due to their unstable performance or the difficulty in seed production. According to the data obtained from the National Agro-Tech Extension and Service Center (http://www.natesc.org.cn), by 2018, a total of 17 varieties have been cultivated to an area of more than 10,000 ha in 9 provinces in China (Table 4), among which Zhejiang province has the largest area, followed by Liaoning province (Fig. 4).

**Table 4:** List of varieties with an area of more than 100,000 ha

Varieties	Female	CMS type	Male	Area(10^4^ ha)	Type	Subspecies	Year of released
Yongyou9	Yongjing2	BT	K6093	71.5	Three-lines	*Japonica*	2007
E jing za 1	N5088S	BT	R187	61.3	Two-lines	*Japonica*	1995
Li you 57	LimingA	BT	C57	59.9	Three-lines	*Japonica*	1980
9 you 418	Xu9201A	BT	C418	40.8	Three-lines	*Japonica*	2000
Yong you 6	Yongjing2A	BT	K4806	28.9	Three-lines	*Japonica*	2005
Han you xiang qing	HanfengA	BT	Xiangqing	25.7	Three-lines	*Japonica*	1989
Yong you 12	Yongjing2A	BT	F5032	25.7	Three-lines	*Japonica*	2010
Yong you 1	Ning67A	BT	K1722	20.7	Three-lines	*Japonica*	2000
Xiu you 57	XiulingA	BT	C57	20.4	Three-lines	*Japonica*	1984
Wan dao 26	7001S	BT	Xiushui04	19.3	Two-lines	*Japonica*	1994
Wan dao 34	80-4A	BT	HP121	13.9	Three-lines	*Japonica*	1996
Yong you 538	Yongjing3A	BT	F7538	13.7	Three-lines	*Indica-japonica*	2013
III you98	MH2003A	BT	R18	13.4	Three-lines	*Japonica*	2002
E jing za 3	N5088S	BT	Minhui128	13.3	Two-lines	*Japonica*	2004
Yong you 17	Yongjing4A	BT	Yonghui12	11.1	Three-lines	*Indica-japonica*	2012
Chun you 59	Chunjiang16A	BT	CH59	10.7	Three-lines	*Japonica*	2009

In the mid-1980s, the total area of *japonica* hybrid rice cultivation was only about 133,000 ha and accounted for just 2% of the total area of *japonica* rice. Even in northern China, the land used for growing hybrid *japonica* rice accounted for only approximately 6% of the total area of *japonica* rice (Xie et al. 2007). By the early 1990s, as studies on hybrid *japonica* rice began to decline, the planting area decreased to 80,000 ha, constituting only about 1% the of *japonica* rice area. However, in recent years, the area used for growing hybrid *japonica* rice has increased and is mainly distributed throughout Liaoning, Jiangsu, Shanghai, Zhejiang, Yunnan, and other provinces (Jiang et al. 2014; Kang 2013; Ma et al. 1998; Ni et al. 2001; Quan et al. 2000). For example, Tianlongyou 619, released by Liaoning Province Crop Variety Approval Committee in 2014 and by National Crop Variety Approval Committee in 2016, developed by a cross of a sterile line featuring more panicles and a restorer line characterized by larger panicles. Tianlongyou 619 is long-grain fragrant rice of excellent quality (“Grade I” quality according to the national standard). This variety provides grain yield of more than 9000 kg/ha, has broad adaptability, and is widely planted in Southern Jilin, Liaoning, Tianjin, Ningxia, Jiangsu, Hainan, and other provinces. The Jiangsu Academy of Agricultural Sciences developed a hybrid *japonica* variety 95You161 by a cross of *japonica* sterile line 95122A and restorer line R161. 95You161 exhibits high quality (“Grade II”) and is grown on 13,400 ha. In 2008, the Shanghai Academy of Agricultural Sciences has developed a hybrid *japonica* variety Huayou 14, which was being used as a typical high-yield variety for the past 3 yrs. Huayou 14 exhibits high “Grade I” quality and is cultivated on an area of 80,000 ha. In the middle-lower Yangtze region, a number of hybrid *japonica* rice varieties with super-high yield and strong heterosis have been developed using *indica-japonica* complementation. Since 2011, a series of new *indica*-*japonica* hybrid rice varieties, including Yongyou and Chunyou, have shown high yield potential by reaching yield levels similar to those of super-high-yielding varieties (Jiang et al. 2014; Kang 2013). Zhongchunyou 84, a subspecies hybrid rice variety with a super-high yield, was developed by a cross between the early-blooming, sterile, dwarf *japonica* line Chunjiang 16A and an *indica*-*japonica* restorer line C84 with wide-compatibility. From 2014 to 2015, Zhongchunyou 84 was the dominant rice variety in Zhejiang Province and was grown on an area of 46,700 ha (Dong et al. 2016). Although the planting area of hybrid *japonica* varieties has increased in recent years, it still accounts for less than 5% of the total *japonica* planting area (Pu et al. 2015). Due to certain key technical barriers that remain to be solved, the difference in the planting area between hybrid *japonica* and *indica* is still significant.

## Current Status of Genomics in Hybrid *japonica*

With the development of high-throughput sequencing technology, hundreds of agronomically relevant heterotic QTLs affecting the performance of heterozygous genotypes have been mapped. Many QTLs contribute to heterosis by dominant or overdominant effects, and some exhibit strong heterotic effects on important agronomical characteristics such as grain yield and flowering time (Huang 2015; Huang 2016; Li et al. 2016; Lin et al. 2019). At the same time, the molecular mechanism of heterosis was gradually elucidated by transcriptome sequencing and genome resequencing technique. In 2015, DNA sequencing of 1495 elite hybrid rice varieties and their inbred parental lines was performed. Comprehensive analyses of heterozygous genotypes revealed that heterosis results mostly from the accumulation of numerous superior alleles with positive dominant effects (Huang et al. 2015). Huang et al. (2016) generated the sequences and recorded the phenotypes of 10,074 F_2_ lines from 17 representative hybrid rice crosses. They documented that a small number of genomic loci from female parents are responsible for a large part of the yield advantage that hybrids have over their male parents. For some of those loci, they found support for partial dominance of heterozygous locus for yield-related traits when all grain-yield traits were considered together. In the process of hybrid rice breeding, breeders tend to introduce different introgressed exogenous genomes unconsciously to shaped heterotic loci in the hybrid rice. Lin et al. (2020) generated two populations of rice F_1_ hybrids using commercial hybrid parents and genotyped the parents by a 50 k SNP chip and genome resequencing, the results from the analysis revealed that the male and female parents have different levels of genome introgressions from other rice subpopulations, including *indica*, *aus*, and *japonica*, therefore shaping heterotic loci in the hybrids. Among the introgressed exogenous genome, heterotic loci, including *Ghd8/DTH8*, *Gn1a*, and *IPA1* existed in wild rice, but were significantly divergently selected among the rice subpopulations, suggesting these loci were subject to environmental adaptation. During modern rice hybrid breeding, heterotic loci were further selected by removing loci with negative effect and fixing loci with positive effect and pyramid breeding. These findings may facilitate future breeding of improved varieties of hybrid rice (Lin et al. 2020). However, these experimental studies utilized mostly *indica* hybrid rice or their parents, while the understanding of the genomics of hybrid *japonica* rice is still lagging behind.

## Problems and Prospects

Under the leadership of Academician Longping Yuan and the joint efforts of several rice-breeding institutes in China, remarkable progress was made in the development of hybrid *japonica* rice. However, some problems restricting further improvements remain in place, necessitating collaborative research. First, the yield advantage of hybrid *japonica* rice is not high enough in comparison with traditional *japonica* rice. On the one hand, due to the limited knowledge of the genetic background of the parents of *japonica* hybrid rice, the genetic distance between the parents of *japonica* hybrid rice is not sufficiently large, leading to a weak yield heterosis. On the other hand, the introduction of the *indica* genome into hybrid *japonica* rice was typically considered as a strategy to enhance the heterosis. However, under the influence of the *indica* genetic background, hybrid *japonica* rice often exhibit premature senescence and differentiation between strong and weak (filled and partially-filled) grains. In addition, with low temperature present during the later growth stages, the weak grains are not sufficiently filled and do not mature fully, reducing the yield potential. Therefore, to avoid the negative effects of the introduction of *indica* rice genetic background, further research should focus on the mechanism of the formation of filled and partially-filled grains and the inheritance of cold tolerance. Second, despite the important breakthroughs and developments in mechanized seed production accomplished by the NJETC and the Shanghai Academy of Agricultural Sciences, the low yield of hybrid *japonica* seeds is the key limiting factor in the applications of hybrid *japonica*. Due to the fact that the restore genes were derived mostly from *indica* rice, the restorer lines with some *indica* background have early blooming time. However, most *japonica* sterile lines exhibit late blooming time and low percent of stigma exposure. Moreover, with the development of directly seeded rice, the need for an increased amount of seeds created a new challenge for the production of hybrid *japonica* rice seeds. Therefore, it is necessary to accelerate germplasm screening and gene mining, and utilize these data to achieve early blooming time, high stigma exposure rate, large stigma size, and strong stigma vigor.

It should also be noted that the growth and development of hybrid *japonica* rice are different from conventional *japonica* rice. Cultivation techniques should be developed according to the characteristics of hybrid *japonica* rice. The hybrid *japonica* rice possesses larger panicles and more grains, and it was usually faced with large sink but small source. In terms of cultivation, an adequate population structure should be established in the early and middle stages of growth to ensure sufficient and effective number of panicles and spikelets, and to avoid the overgrowth of plants. Attention should be given to ensuring grain filling and preventing premature senescence by postponing panicle fertilizer. To fully utilize the yield potential of hybrid *japonica* rice, early sowing and transplanting should be implemented to ensure that the heading and grain-filling occur at the most opportune period.

Finally, the cooperation between scientific research institutions and seed enterprises should be enhanced to jointly promote the commercial operation and industrial development of hybrid *japonica* rice, accelerate the expansion and application of new hybrid japonica rice varieties, and seize the advantages of hybrid *japonica* rice such as reduced use of fertilizer and water, strong resistance, and high yield. These steps will improve the utilization of middle- and low-yield fields and ensure food security.

## Conclusions

With the increasing demand for high-quality *japonica* rice, the prospects for the development of hybrid *japonica* rice are increasingly better, particularly in China. Although the number and the spread area of hybrid *japonica* rice varieties lag behind hybrid *indica*, the research on hybrid *japonica* rice progressed remarkably over the past 70 years. Several male sterile lines (e.g., Dian-type, BT-type, WA -type, and Yinshui-type) with their corresponding restorer and maintainer lines have been used in the three-line system of growing *japonica* hybrid rice. The development of photo-thermo-sensitive genic male sterile lines for the two-line system also promises great potential for improving grain yield in hybrid *japonica* rice. Meanwhile, remarkable progress has been made in research on molecular mechanisms for heterosis, stigma exposure rate, flowering time, and male sterility in hybrid *japonica*. In future, exploiting and pyramiding the superiority genes with yield-related genes by MAS will have an important role in increasing grain yield of hybrid *japonica*. Given the present limitations, we have proposed four effective strategies to develop hybrid *japonica*: (1) increasing parental genetic distance and introducing the wide-compatibility genes; (2) accelerating germplasm screening, gene mining, and utilizing the data to achieve early blooming time, high stigma exposure rate, large stigma size, and strong stigma vigor; (3) using new cultivation techniques specific for hybrid *japonica*; and (4) enhancing cooperation with extension departments and cooperatives.

## Data Availability

Not applicable.
